# Comparative Study of Amide Proton Transfer Imaging and Intravoxel Incoherent Motion Imaging for Predicting Histologic Grade of Hepatocellular Carcinoma

**DOI:** 10.3389/fonc.2020.562049

**Published:** 2020-10-29

**Authors:** Baolin Wu, Fei Jia, Xuekun Li, Lei Li, Kaiyu Wang, Dongming Han

**Affiliations:** ^1^Department of Radiology, Huaxi MR Research Center (HMRRC), Functional and Molecular Imaging Key Laboratory of Sichuan Province, West China Hospital of Sichuan University, Chengdu, China; ^2^Department of Magnetic Resonance, The First Affiliated Hospital of Xinxiang Medical University, Xinxiang, China; ^3^MR Research China, GE Healthcare, Beijing, China

**Keywords:** hepatocellular carcinoma, amide proton transfer, intravoxel incoherent motion, histologic grade, prediction

## Abstract

**Background:** Preoperative grading of hepatocellular carcinoma (HCC) is an important factor associated with prognosis after liver resection. The promising prediction of the differentiation of HCC remains a challenge. The purpose of our study was to investigate the value of amide proton transfer (APT) imaging in predicting the histological grade of HCC, compared with the intravoxel incoherent motion (IVIM) imaging.

**Methods:** From September 2018 to February 2020, 88 patients with HCC were enrolled and divided into four groups (G1, G2, G3, and G4) based on the histologic grades. Preoperative APT signal intensity (SI), apparent diffusion coefficient (ADC), true molecular diffusion coefficient (D), pseudo-diffusion coefficient (D^*^), and perfusion fraction (*f* ) of HCC were independently measured by two radiologists. The averaged values of those parameters were compared using an analysis of variance. The Spearman rank analysis was used to compare the correlation between those imaging parameters and the histological grades. Receiver operating characteristic (ROC) curve analysis was used to explore the predictive performance.

**Results:** There were significant differences in APT SI, ADC, D, and *f* among the four grades of HCC (all *P* < 0.001). A moderate to good relationship was found between APT SI and the histologic grade of HCC (*r* = 0.679, *P* < 0.001). APT SI had an area under the ROC curve (AUC) of 0.890 (95% CI: 0.805–0.947) for differentiating low- from high-grade HCC, and the corresponding sensitivity and specificity were 85.71% and 82.05%, respectively. Comparison of ROC curves demonstrated that the AUC of APT SI was significantly higher than those of IVIM-derived parameter (*Z* = 2.603, *P* = 0.0092; *Z* = 2.099, *P* = 0.0358; *Z* = 4.023, *P* = 0.0001; *Z* = 2.435, *P* = 0.0149, compared with ADC, D, D^*^, and *f* , respectively). Moreover, the combination of both techniques further improved the diagnostic performance, with an AUC of 0.929 (95% CI: 0.854–0.973).

**Conclusion:** APT imaging may be a potential noninvasive biomarker for the prediction of histologic grading of HCC and complements IVIM imaging for the more accurate and comprehensive characterization of HCC.

## Introduction

Hepatocellular carcinoma (HCC) is the most common primary liver cancer, and its incidence has been increasing worldwide in recent decades (1). Notably, frequent tumor recurrence is observed in some cases after liver resection (2). After hepatic resection, patients with early tumor recurrence have a worse survival rate than those with late tumor recurrence (3). It has been reported that histological grading of HCC is one of the most important predictive factors for early tumor recurrence and prognosis after curative liver resection (4). Thus, to select the optimal therapeutic strategy and help to direct the proper management of HCC patients, it will be of great clinical significance to develop a more accurate tool to evaluate the histologic grade of HCC prior to liver resection.

With the development of diagnostic imaging techniques, especially advanced magnetic resonance (MR) imaging, many researchers have attempted to develop useful and noninvasive imaging biomarkers for the grading of HCC. Recently, conventional diffusion-weighted imaging (DWI) and intravoxel incoherent motion (IVIM) diffusion-weighted MR imaging (5–7) have shown their potential values in providing useful biomarkers for the prediction of HCC grading. The IVIM model, a common MR imaging technique, can obtain additional quantitative parameters that describe water diffusivity, perfusion (pseudo diffusion coefficient), and the perfusion fraction of tissues compared with conventional DWI. In recent years, the IVIM approach has been one of the most widely used MR imaging techniques to evaluate the histologic grade of HCC (6, 8).

Chemical exchange saturation transfer imaging (CEST) is one of the routine magnetization transfer techniques, which can be used to detect the characteristics of the mobile protein and amino acid in human body (9). Amide proton transfer (APT) imaging was developed as one of the CEST imaging techniques (10) and mainly measures the chemical transfer properties of amide protons located at the +3.5 ppm, thus enabling indirect determination of cellular mobile protein and peptide levels (11). Recently, investigators have attempted to study the potential value of APT imaging in estimating the histological grade of tumor. In a recent prospective study (12), APT imaging-guided stereotactic biopsy was performed in patients with gliomas, and the authors found that the APT SIs in the high-grade specimens were significantly higher than those in the low-grade specimens. Additionally, the APT imaging has also been applied to endometrioid endometrial adenocarcinoma (EEA) (13) and squamous cell carcinoma of the cervix (SCCC) (14) to evaluate the tumor characteristics. As a novel contrast mechanism in the field of molecular imaging, APT imaging has provided new diagnostic ideas for the grading of HCC. A recent study has revealed that both APT imaging and DWI had good diagnostic performance in differentiating high- from low-grade of HCC, indicating that APT imaging may be a useful imaging biomarker that complements DWI for the more accurate and comprehensive HCC characterization (15).

Although both the IVIM and APT imaging have been used to evaluate the histologic grade of HCC, the application of APT imaging on HCC is still limited, and whether APT imaging can provide a better diagnostic performance for differentiating HCC grades than IVIM-derived parameters has not been fully understood. Thus, the purpose of the present study was to investigate the utility of APT imaging for evaluating the histologic grade of HCC, compared with the IVIM-derived parameters.

## Materials and Methods

### Patients

This prospective study was approved by the institutional review board and followed the ethical guidelines of the Declaration of Helsinki, and written informed consent was acquired from each subject before inclusion. From September 2018 to February 2020, 127 patients with suspected HCC based on clinical history and/or previous ultrasonography and/or CT were initially enrolled and underwent preoperative liver MR imaging. Patients were included based on the following criteria: (1) with primary HCC lesions that had not been previously treated; (2) aged ≥18 years; (3) had no contraindication to MR examinations; (4) did not undergo any form of contrast-enhanced examination 24 h before APT and IVIM imaging.

Thirty-nine patients were excluded due to various reasons: (1) did not undergo surgery and/or histopathological examination (*n* = 17); (2) had a history of preoperative treatment prior to MR imaging, such as radiofrequency ablation, transarterial chemoembolization, percutaneous ethanol injection, or a combination of these (*n* = 9); (3) were not diagnosed with HCC after evaluating the final histopathological examinations (*n* = 7); (4) had a low quality of MR images (*n* = 3); and (5) had tumor lesions smaller than 1 cm (*n* = 3). Eighty-eight patients (78 males and 10 females; mean age 53.45 ± 13.67 years, range from 31 to 67 years) with histopathological-confirmed HCC were finally included. The etiology of liver disease included hepatitis B virus (*n* = 74), hepatitis C virus (*n* = 10), and others (*n* = 4). The mean tumor size was 7.82 ± 3.65 cm. According to the major Edmondson and Steiner grading system on the final pathologic reports, all the tumors were histologically classified as follows: grade 1 (G1, *n* = 19), grade 2 (G2, *n* = 30), grade 3 (G3, *n* = 28), and grade 4 (G4, *n* = 11). Furthermore, G1 and G2, and G3 and G4 were defined as low- and high-grade HCC, respectively, based on the evidence that significant differences in long-term survival were demonstrated between low- and high-grade HCC (16, 17).

### Data Acquisition

All patients underwent liver MR scanning on a 3T device (GE DISCOVERY MR750; GE Healthcare, Milwaukee, Wisconsin, USA) with a 32-channel phased-array torso coil. The scans ranged from the top of the diaphragm to the lower edge of the liver. All patients were instructed to fast and abstain from food and water for 6–8 h prior to MR examinations, and the patients were also trained in the techniques of even breathing and breath-holding. First, conventional liver MR images were obtained using an axial respiratory-triggered fat-suppressed fast spin-echo T_2_-weighted imaging sequence and a three-dimensional Liver Acquisition with Volume Acceleration-Flexible (LAVA-Flex) sequence with breath-hold. The total scanning time of the conventional MR imaging was ~7 min. After conventional MR scanning, two-dimensional axial APT imaging was performed using a single-shot fast spin echo-planar imaging sequence with free-breathing (Fermi pulses, with a power level of 2 μT and a total saturation duration of 2 s for four multiple pulses). To obtain an APT z-spectrum, the APT imaging was repeated at 49 saturation frequency offsets (from 600 to −600 Hz with an interval of 25 Hz). In addition, three unsaturated images at the offset of 5,000 Hz were also acquired for signal normalization. Specifically, the “frequency of 5000 Hz”, i.e., 39 ppm (1 ppm = 128 Hz on 3 T), is far away from water in 4.7 ppm and other metabolites (±12 ppm) that can have CEST effect. Therefore, there will not be any “CEST-effect” when using saturation pulse at the frequency offset of 5,000 Hz, and this z-spectrum can be taken as water signal S0 for calculation. The APT images were acquired through a single section that was selected as the one showing the maximum tumor area according to axial T_2_-weighted imaging, and the total acquisition time of APT imaging was 2 min and 10 s. Acquisition of IVIM was performed by using a spin-echo echo-planar imaging sequence with free-breathing, and the *b* values were 0, 20, 40, 80, 160, 200, 400, 600, 800, and 1000 s/mm^2^. The total scanning time for IVIM imaging was 5 min. Detailed MR imaging parameters are summarized in Table 1.

**Table 1:** MRI parameters.

**Parameters**	**T_**1**_-weighted imaging**	**T_**2**_-weighted imaging**	**APT imaging**	**IVIM imaging**
Sequences	Axial LAVA flex	Axial FSE T2WI	Axial CEST-EPI	Axial SE-EPI
Repetition time/echo time (ms)	4.3/1.6	10,000/70	2500/11.9	2500/58.8
Flip angle (°)	14	110	20	90
Field of view (mm^2^)	360 × 324	360 × 360	400 × 400	380 × 380
Matrix (frequency × phase)	260 × 210	320 × 320	128 × 128	128 × 128
Number of excitations	1	1.5	1	2–6
Slice thickness (mm)	4.0	4.0	5.0	5.0
Slice gap (mm)	0	0.5	N/A	1.0
No. of slices	24	24	1	20

*LAVA, liver acquisition with volume acceleration; CEST, chemical exchange saturation transfer; FSE, fast spin-echo; SE, spin-echo; EPI, echo-planar imaging; N/A, not applicable*.

### Data Analysis

In CEST imaging, the magnetic transfer ratio (MTR) was defined as 1 – *S*_*sat*_/*S*_0_, where *S*_0_ and *S*_*sat*_ are the water signals before and after pulse saturation, respectively (10). For APT imaging, the asymmetry analysis at 3.5 ppm downfield from the water signal was calculated as MTR_asym_ (3.5 ppm): MTR_asym_ (3.5 ppm) = *S*_*sat*_ (−3.5 ppm)/*S*_0_ –* S*_*sat*_ (+3.5 ppm)/*S*_0_ = MTR'_asym_ (3.5 ppm) + APTR, where MTR'_asym_ is the inherent asymmetry of the conventional magnetization transfer effect and APTR is the APT ratio (10). As a result, the measured MTR_asym_ (3.5 ppm) values can be defined as the apparent APT SIs, and therefore, it is appropriate to define the calculated MTR_asym_ (3.5 ppm) images as APT-weighted imaging. In our study, the APT SI was defined as MTR_asym_ (3.5 ppm) × 100 (%). The detailed analysis methods of the parameters derived from APT and IVIM imaging have been described previously (13, 18). After data acquisition, the images were transferred to a GE AW4.6 workstation (Advantage workstation 4.6; GE Healthcare, Milwaukee, Wisconsin, USA) and data analysis was performed independently by two observers (observer 1, B.L.W., and observer 2, F.J., with 7 and 5 years of experience in liver MR imaging, respectively) who were blinded to the histopathological results. Borders were drawn along the edge of the tumor on the original images of the IVIM and APT sequences by referring to the conventional T_1_- and T_2_-weighted images. On the largest diameter of each lesion, three regions of interest (ROIs) with the same size (~100 mm^2^) were manually delineated in the solid part of the tumor, carefully avoiding the edge of the tumor and areas of cystic degeneration, necrosis, and bleeding. The ROIs were automatically copied to the APT and IVIM pseudo-colored maps to obtain the mean APT SI, apparent diffusion coefficient (ADC), true molecular diffusion coefficient (D), pseudo-diffusion coefficient (D^*^), and perfusion fraction (*f* ) values for each ROI. We calculated the averaged values of the three ROIs for each parameter, and the averaged values calculated by the two observers were recorded for further analysis. In patients with multifocal lesions, the lesion with the largest diameter was chosen for analysis.

### Pathologic Evaluation

The pathologic evaluation was performed for the surgically resected specimens from the 88 patients by a pathologist (a non-author with 31 years of experience in liver pathology) who was blinded to the liver MR imaging results. Three aspects were included in the pathologic reports: the histologic grade, size, and location. Based on the Edmondson and Steiner grading system (19), the major (predominant grade within the tumor) and worst (grade of the most poorly differentiated region) histologic grade of HCC was reported. One of the authors (X.K.L.) was responsible for the collection of pathologic data.

### Statistical Analysis

A Shapiro–Wilk test was used to evaluate the normal distribution of the imaging parameters. Then, if those parameters were determined to be normally distributed, we calculated the intraclass correlation coefficient (ICC), a measurement reflecting the differences in reliability between the two independent observers, to assess the reproducibility of those imaging parameters: excellent agreement (ICC ≥ 0.75), good agreement (0.60 ≤ ICC ≤ 0.74), fair agreement (0.40 ≤ ICC ≤ 0.59), and poor agreement (ICC < 0.40) (13). The differences in APT SI and IVIM-derived parameters among different HCC grades were analyzed using an analysis of variance (ANOVA), followed by a *post-hoc* test using least significant difference method. The Spearman rank analysis was used to compare the correlation between those imaging parameters and the histological grades. The correlation coefficient, rho (*r*), was obtained to compare the degree of the correlations as follows: little or no relationship (0 ≤ *r* < 0.25), fair (0.25 ≤ *r* < 0.5), moderate to good (0.5 ≤ *r* < 0.75), and very good to excellent (*r* ≥ 0.75) (6). A Bland–Altman plot analysis was used to illustrate the agreement between the interobserver measurements, receiver operating characteristic (ROC) analyses were performed to evaluate the diagnostic performance of the APT SI and IVIM-derived parameters in distinguishing the low-grade (G1 and G2) and high-grade (G3 and G4) HCC, and the optimal cutoff values and the corresponding sensitivity and specificity values were calculated. Delong test (20) was used for the comparison of ROC curves. Statistical analyses were performed using SPSS software version 22.0 (IBM SPSS Statistics, Armonk, NY) and MedCalc software version 19.2.0 (MedCalc, Mariakerke, Belgium). A *P* < 0.05 was considered to indicate a significant difference.

## Results

### Interobserver Agreement

**Figure 1:**
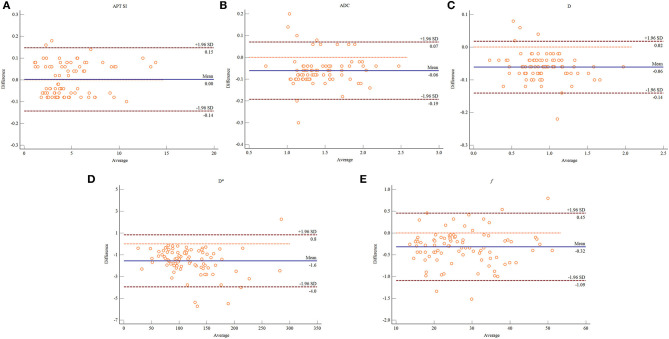
Bland–Altman plots showing the distribution of the differences of the APT SI **(A)**, ADC **(B)**, D **(C)**, D* **(D)**, and *f* **(E)** between the two observers. The dark blue horizontal solid line represents the mean difference, and the two dark red horizontal lines represent the limits of agreement, which are defined as the mean difference plus or minus 1.96 times the standard deviation of the differences.

The Shapiro–Wilk test revealed that all the quantitative imaging parameters measured by the two observers were normally distributed (observer 1: *P* = 0.124 for APT SI, 0.167 for ADC, 0.247 for D, 0.089 for D^*^, and 0.234 for *f* ; observer 2: *P* = 0.215 for APT SI, 0.221 for ADC, 0.283 for D, 0.142 for D^*^, and 0.198 for *f* ). The ICCs between the two observers were 0.998 [95% confidence interval (CI): 0.996–0.998], 0.989 (95% CI: 0.983–0.993), 0.995 (95% CI: 0.993–0.997), 0.994 (95% CI: 0.990–0.996), and 0.996 (95% CI: 0.994–0.998) for APT SI, ADC, D, D^*^, and *f* , respectively, suggesting an excellent reliability. The Bland–Altman analysis of the APT SI and IVIM-derived parameters measured by the two observers showed good concordance, with at most five values beyond the 95% limits of agreement (Figure 1).

### Comparisons of APT SI and IVIM-Derived Parameters

**Figure 2:**
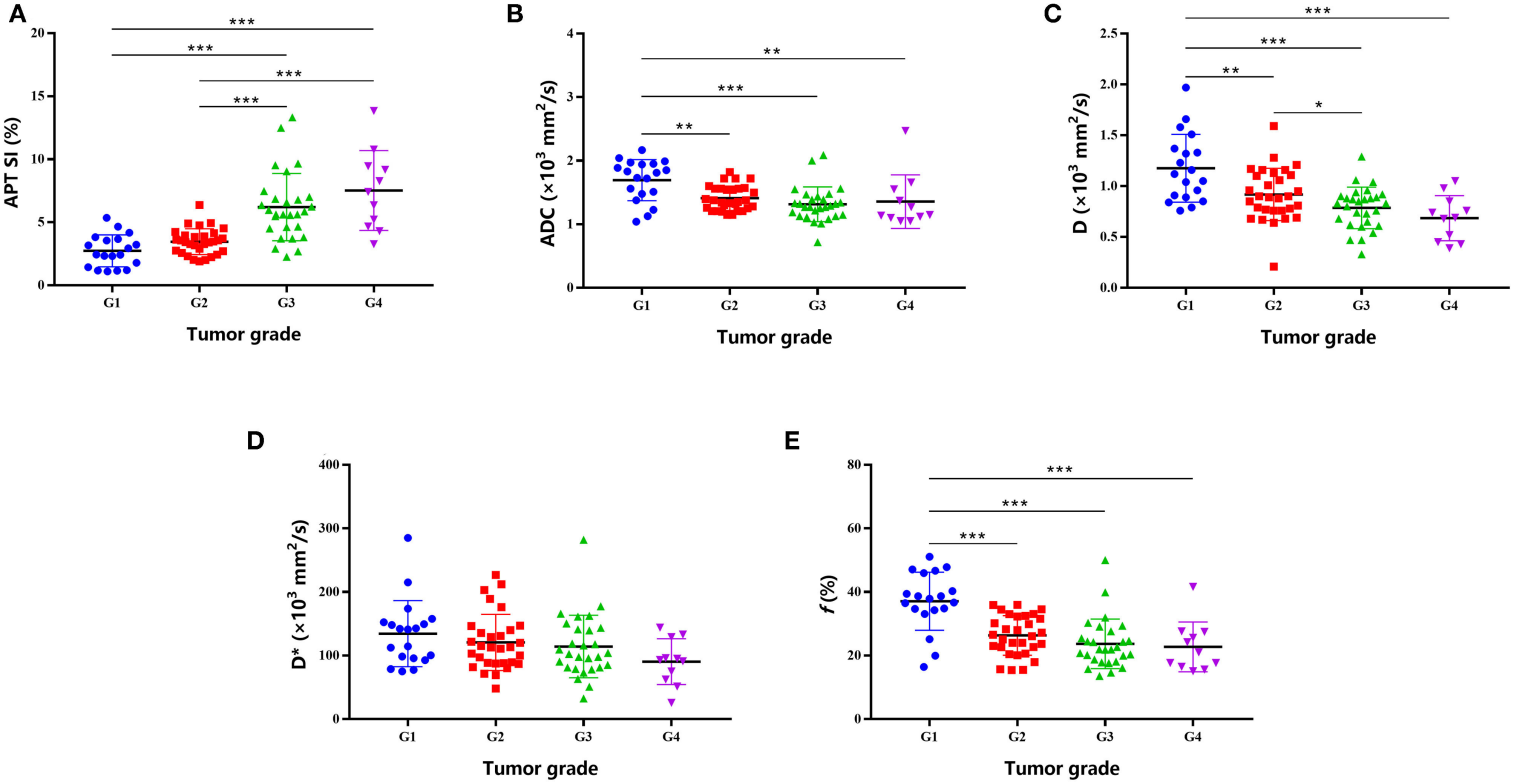
Plots show individual data points, averages (transverse lines), and standard deviations (vertical lines) of APT SI **(A)**, ADC **(B)**, D **(C)**, D* **(D)**, and *f* **(E)** for each HCC grade. *P < 0.05; **P < 0.01; ^***^P < 0.001 for pairwise comparisons.

**Figure 3:**
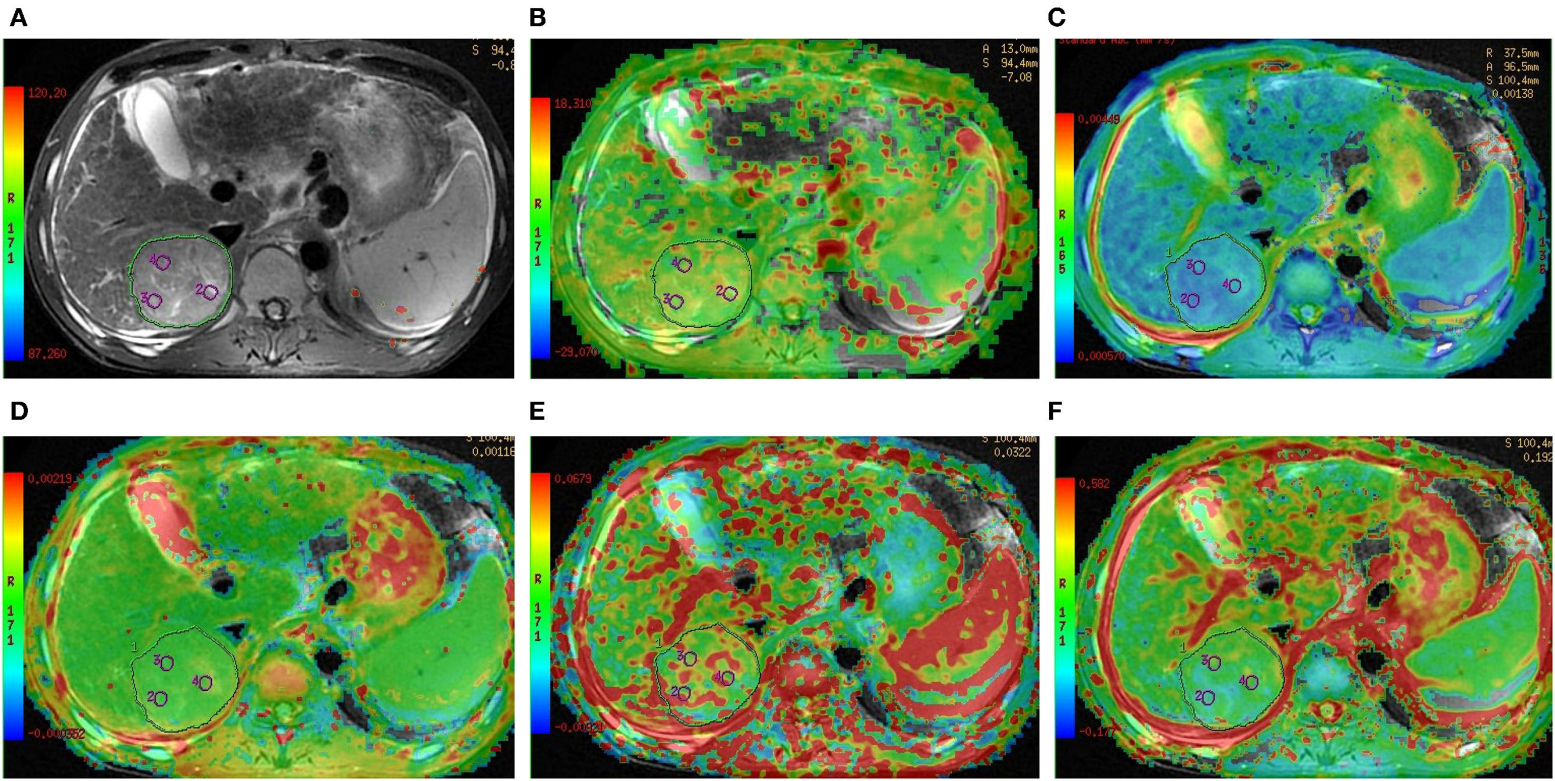
Preoperative MR images of one lesion in a 52-year-old man with grade 2 HCC. **(A)** T_2_-weighted image, **(B)** APT image of HCC fused with T_2_-weighted imaging indicates that the average APT SI value determined by two observers is 3.17%, **(C–F)** IVIM-derived pseudo-colored maps of HCC (ADC, D, D*, and *f* , respectively) fused with T_2_-weighted imaging indicate that the average ADC, D, D*, and *f* values determined by two observers were 1.54 × 10^−3^ mm^2^/s, 1.59 × 10^−3^ mm^2^/s, 100.18 × 10^−3^ mm^2^/s, and 34.55%, respectively.

**Figure 4:**
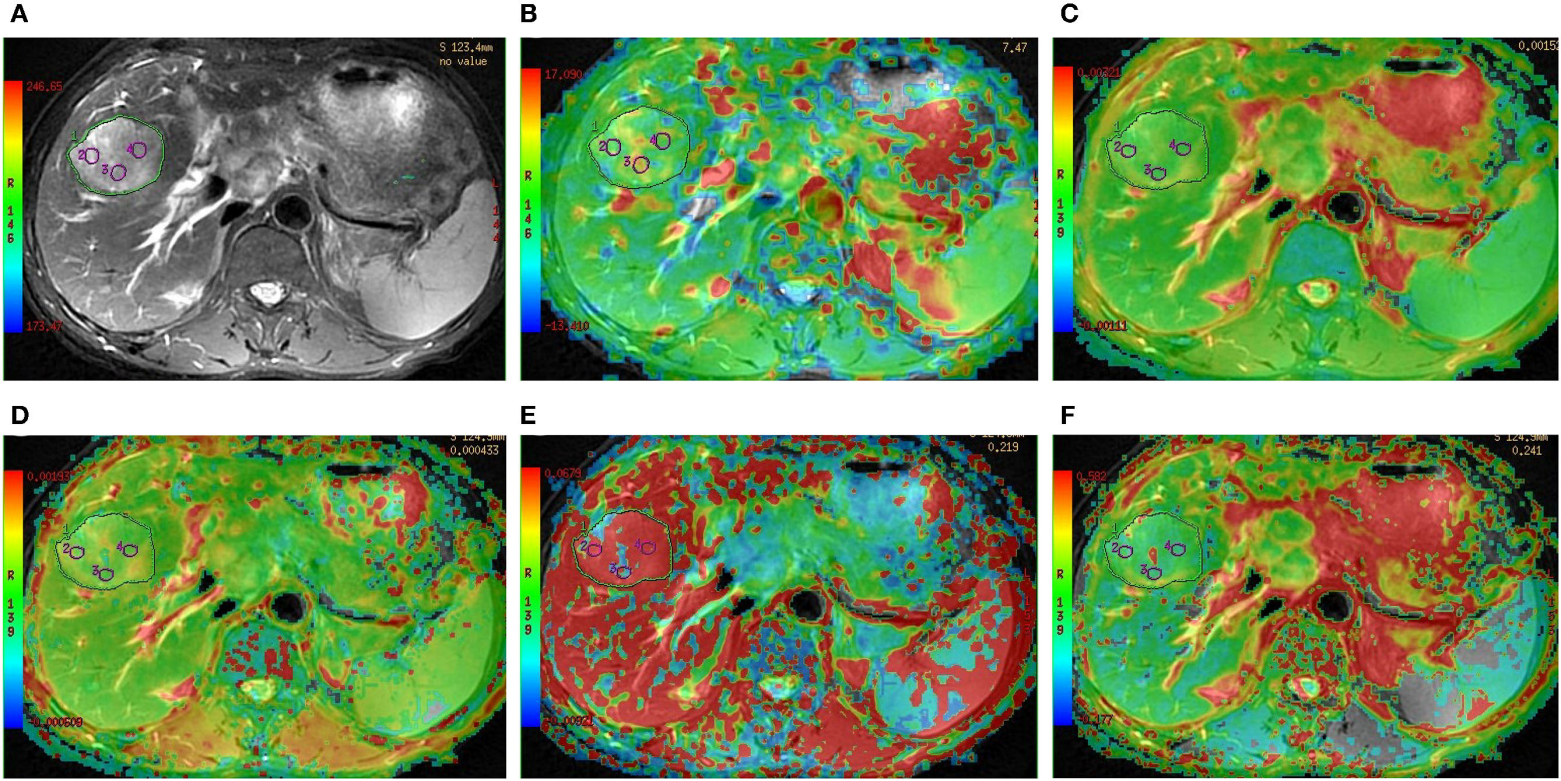
Preoperative MR images of one lesion in a 47-year-old man with grade 4 HCC. **(A)** T_2_-weighted image, **(B)** APT image of HCC fused with T_2_-weighted imaging indicates that the average APT SI value determined by two observers is 6.35%, **(C–F)** IVIM-derived pseudo-colored maps of HCC (ADC, D, D*, and *f* , respectively) fused with T_2_-weighted imaging indicate that the average ADC, D, D*, and *f* values determined by two observers were 1.14 × 10^−3^ mm^2^/s, 0.45 × 10^−3^ mm^2^/s, 98.41 × 10^−3^ mm^2^/s, and 27.52%, respectively.

As shown in Table 2 and Figure 2, there were significant differences in APT SIs, ADC, D, and *f* values among different tumor grades. Pairwise comparisons revealed significant differences in APT SIs between G1 and G3, G1 and G4, G2 and G3, and G2 and G4; and in D between G1 and G2, G1 and G3, G1 and G4, and G2 and G4; and in ADC between G1 and G2, G1 and G3, and G1 and G4; and in *f* between G1 and G2, G1 and G3, and G1 and G4 (all *P* < 0.05). Figures 3, 4 show MR images of two patients with low- and high-grade HCC, respectively.

**Table 2:** Comparisons of the APT- and IVIM-derived parameters among different histological grades of HCC.

	Edmondson–Steiner Grade*				
**Parameters**	**G1 (*n* = 19)**	**G2 (*n* = 30)**	**G3 (*n* = 28)**	**G4 (*n* = 11)**	***P* value**
APT SI (%)	2.74 ± 1.27	3.47 ± 1.02	6.21 ± 2.66	7.53 ± 3.17	<0.001
ADC (× 10^−3^ mm^2^/s)	1.70 ± 0.32	1.41 ± 0.19	1.31 ± 0.27	1.35 ± 0.42	<0.001
D (× 10^−3^ mm^2^/s)	1.18 ± 0.33	0.92 ± 0.26	0.79 ± 0.20	0.69 ± 0.22	<0.001
D* (× 10^−3^ mm^2^/s)	134.42 ± 51.93	120.72 ± 44.18	114.24 ± 49.18	90.53 ± 35.98	0.101
*f* (%)	37.13 ± 9.13	26.35 ± 6.24	23.69 ± 7.79	22.74 ± 7.84	<0.001

* *Data are expressed as mean ± standard deviation*.

### Correlation of Histologic Grade With APT SI and IVIM-Derived Parameters

The Spearman correlation coefficients between the parameters derived from APT and IVIM imaging and the histopathological grades of HCC are shown in Table 3. A moderate to good relationship was found between APT SI and the histologic grade of HCC (*r* = 0.679, *P* < 0.001). There was also a moderate to good relationship between the histologic grade of HCC and D and *f* (*r* = −0.517, *P* < 0.001 and *r* = 0.502, *P* < 0.001, respectively). A fair relationship was demonstrated between the histologic grade of HCC and ADC (*r* = −0.433, *P* < 0.001), and a little relationship was found between the histologic grade of HCC and D^*^ (*r* = −0.247, *P* < 0.021).

**Table 3:** Spearman correlation coefficients of the APT- and IVIM-derived parameters with the histopathological grades of HCC.

**Parameters**	**Spearman correlation coefficients**	***P* value**
APT SI (%)	0.679	<0.001
ADC (× 10^−3^ mm^2^/s)	−0.433	<0.001
D ( × 10^−3^ mm^2^/s)	−0.517	<0.001
D* (× 10^−3^ mm^2^/s)	−0.247	0.021
*f* (%)	−0.502	<0.001

### ROC Analysis for Diagnostic Performance of APT SI and IVIM-Derived Parameters

**Figure 5:**
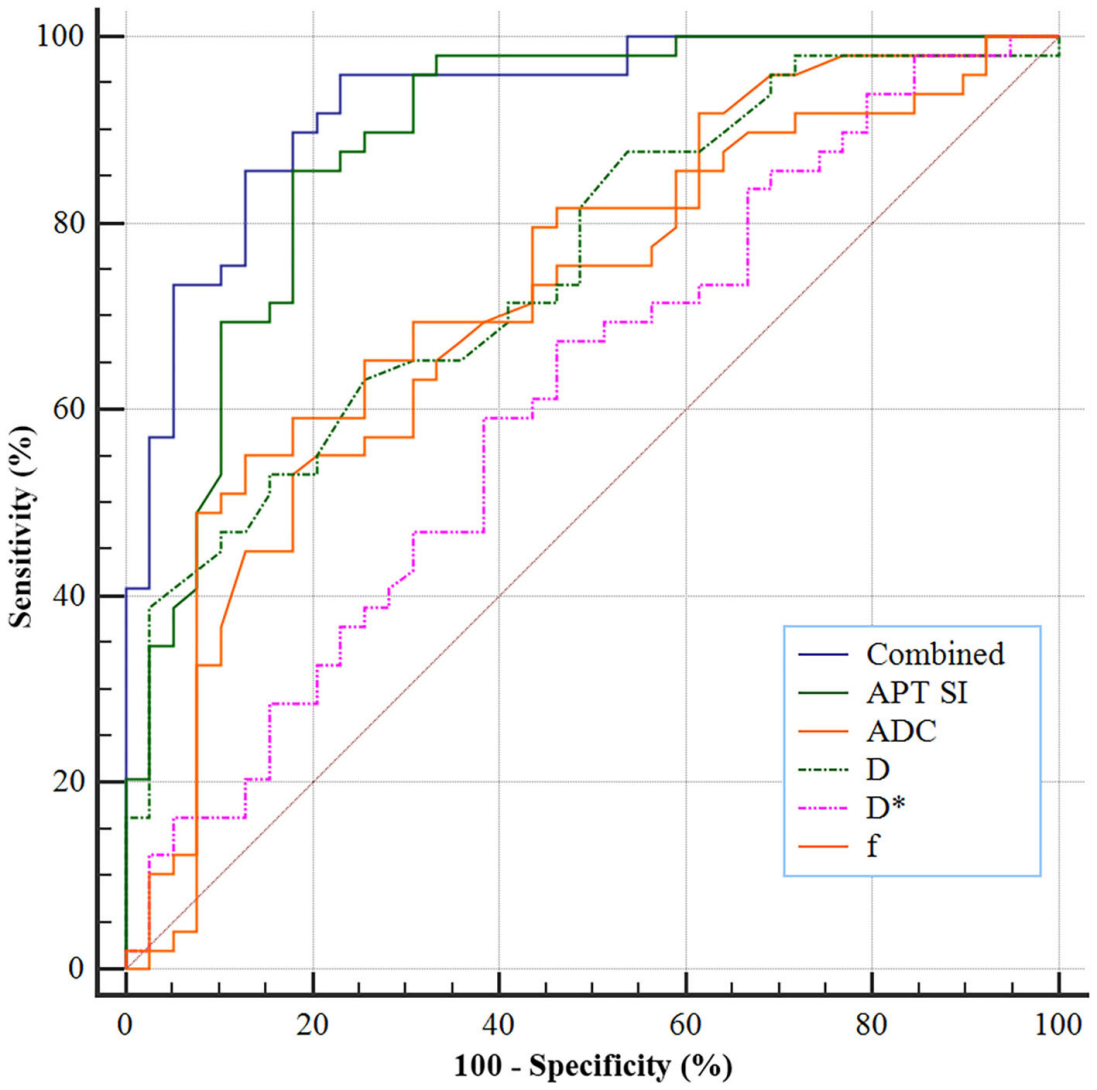
ROC analyses of the APT SI and IVIM-derived parameters for differentiating low- from high-grade HCC. The AUCs for APT SI, ADC, D, D*, and *f* were 0.890, 0.713, 0.757, 0.612, and 0.733, respectively, and a combination of both techniques improves the AUC to 0.929.

As shown in Figure 5, the ROC analyses demonstrated a better diagnostic performance of APT SI [area under the ROC curve (AUC) = 0.890, 95% CI: 0.805–0.947] than IVIM-derived parameters [AUCs for ADC, D, D^*^, and *f* were 0.713 (95% CI: 0.606–0.804), 0.757 (95% CI: 0.654–0.842), 0.612 (95% CI: 0.502–0.714), and 0.733 (95% CI: 0.628–0.822), respectively] for differentiating low- from high-grade HCC. Furthermore, the combination of APT SI and DKI-derived parameters showed an improvement of diagnostic performance, with an AUC of 0.929 (95% CI: 0.854–0.973). Corresponding sensitivity, specificity, and optimal cutoff values are listed in Table 4. Moreover, comparison of ROC curves demonstrated that the AUC of multivariant parameters (APT combined with IVIM) was significantly higher than those of univariant parameters (*Z* = 2.029, *P* = 0.0452; *Z* = 3.428, *P* = 0.0006; *Z* = 3.508, *P* = 0.0005; *Z* = 4.878, *P* < 0.0001; *Z* = 3.302, *P* = 0.0010, compared with APT SI, ADC, D, D^*^, and *f* , respectively), and the AUC of APT SI was significantly higher than those of IVIM-derived parameter (*Z* = 2.603, *P* = 0.0092; *Z* = 2.099, *P* = 0.0358; *Z* = 4.023, *P* = 0.0001; *Z* = 2.435, *P* = 0.0149, compared with ADC, D, D^*^, and *f* , respectively).

**Table 4:** Diagnostic performance of the APT- and IVIM-derived parameters in differentiating the low- from high-grade HCC.

**Parameters**	**AUC (95% CI)**	***P* value**	**Optimal cutoff value**	**Youden index**	**Sensitivity (%)**	**Specificity (%)**
APT SI (%)	0.890 (0.805–0.947)	<0.001	4.31	0.678	85.71	82.05
ADC (× 10^−3^ mm^2^/s)	0.713 (0.606–0.804)	<0.001	1.48	0.351	53.06	82.05
D (× 10^−3^ mm^2^/s)	0.757 (0.654–0.842)	<0.001	0.94	0.377	53.06	84.62
D* (× 10^−3^ mm^2^/s)	0.612 (0.502–0.714)	0.066	97.95	0.212	67.35	53.85
*f* (%)	0.733 (0.628–0.822)	<0.001	29.35	0.423	55.1	87.18
Combined	0.929 (0.854–0.973)	<0.001	-	0.729	85.71	87.18

## Discussion

Our study demonstrated differences in values of preoperative APT SI, D, ADC, and *f* among different histologic grades of HCC. In addition, compared with IVIM-derived parameters, APT SI was more correlated with the histologic grade of HCC (*r* = 0.679, *P* < 0.001). Furthermore, comparisons of the ROC curves showed that the AUC of APT SI was significantly higher than that of each IVIM-derived parameter. Our findings indicate that the APT imaging, a novel molecular MR imaging technique, may be more accurate in differentiating low- from high-grade of HCC than IVIM imaging, with a sensitivity and specificity of 85.71% and 82.05%, respectively.

The potential value of APT imaging in estimating the histologic grades of tumors, such as SCCC (14, 21), diffuse gliomas (22), and EEA (13), has been demonstrated by previous studies. For example, Li et al. investigated the application of APT imaging in estimating histologic grades of SCCC and found that APT SI was positively correlated with the SCCC grades (14). Previous studies have demonstrated a progressive increase of APT SI from low- to high-grade of gliomas and positive correlations between APT SI and Ki-67 LI and between APT SI and cell density (12, 22). Moreover, a positive correlation between the APT SI and the histologic grades of EEA was demonstrated by a recent study (13). Thus, our findings are compatible with these previous studies and indicate that APT imaging may be a promising method for predicting the histologic grades of tumors.

Theoretically, the effect of APT imaging in tumor is primarily correlated with the tissue content of labile amide protons of mobile proteins (23, 24). In the present study, we found that the APT SIs progressively increased from low- to high-grade HCC. In line with our finding, a recent study found that the APT SIs of high-grade HCC were significantly higher than those of low-grade HCC (15). Malignant tumors often show obvious cell and structural atypia, including an increase in the nuclear-to-cytoplasmic ratio, megakaryocytes and malformed nuclei appear, and the number of ribosomes in the cytoplasm also increase (14). As HCC becomes more poorly differentiated during hepatocarcinogenesis, cellular density and nuclear-to-cytoplasmic ratios increase, while the architecture becomes more complicated (25). The association of high APT SI values and high cellularity and proliferation has been clearly demonstrated in brain tumors (11, 12, 26). Therefore, the upward trend in APT SI for high-grade HCC may be associated with several factors, such as a higher tumor cell proliferation rate and cellular density.

Additionally, we found significant differences in D, ADC, and *f* values among different HCC grades, and higher-grade HCC had lower D and ADC values than lower-grade HCC. IVIM imaging can reflect the characteristics of lesions in terms of cell density, microcirculation perfusion, and tissue complexity (18). As reported previously, a decrease in both ADC and D values may be attributed to the increased cellular density, nuclear-to-cytoplasmic ratios, and architectural complications in higher-grade HCC (27). In line with our findings, Zhu et al. reported a downward shift of D and ADC values from low- to high-grade HCC (8).

Furthermore, we found a moderate to good relationship between the histologic grade of HCC and D and *f* (*r* = −0.517, *P* < 0.001 and *r* = 0.502, *P* < 0.001, respectively). A recent study also showed a moderate to good relationship between the histologic grade of HCC and D (8), which is consistent with our study. Further ROC curve analysis revealed that the AUC of D was higher than that of ADC (0.757 vs. 0.713). A recent meta-analysis focusing on the diagnostic accuracy of quantitative diffusion parameters in the pathological grading of HCC has confirmed that the D value was superior to the ADC value for discriminating the HCC grade, which supports our findings (28). However, APT SI showed the highest AUC (AUC = 0.890; 95% CI: 0.805–0.947) in differentiating low- from high-grade HCC, and the AUC of APT SI was significantly higher than those of the IVIM-derived parameters, indicating that APT imaging is more accurate to predict the histologic grade of HCC than IVIM imaging. We further analyzed the additive value of APT to IVIM imaging in the differentiation between low- and high-grade HCC. The results revealed that a combination of both MR imaging techniques (APT and IVIM) could further improve the diagnostic performance. Therefore, our findings suggest that APT imaging is superior to IVIM imaging in the evaluation of HCC characteristics, and a combination of both can provide a more accurate and comprehensive reflection to HCC characteristics.

We acknowledged several limitations of our study. First, the APT imaging was obtained for only one section per patient because of time limitations for the imaging protocol; thus, we could only acquire the imaging parameters on the maximum tumor area. Second, the IVIM and APT sequences are based on EPI acquisition, with low resolution and poor signal-to-noise ratio, which are easily affected by motion and susceptibility artifacts (29). In addition, the IVIM and APT images were acquired with free breathing, resulting in decreased signal-to-noise ratio on parameter maps. However, the free-breathing protocol was recommended in several studies (30, 31) because of its good reproducibility and shorter acquisition time compared with that of respiratory-triggered and breath-hold imaging. Third, the freehand ROI analysis could produce definite artificial errors, which might affect the accuracy of the values of those quantitative imaging parameters. Fourth, the APT imaging can be affected by fat (32), and liver may have higher fat fraction compared to lots of other organs. However, our study did not measure the fat signal of liver due to limited scanning time, and whether there is a linear trend between APT SI and fat signal is unclear. Thus, future studies are needed to clarify this question. Fifth, this is a single-center study with a relatively small sample size. A prospective cohort study with a large sample size is needed in the future to provide more reliable findings.

In summary, our study showed that APT SI was positively correlated with the histologic grading of HCC and had a better diagnostic performance than IVIM-derived parameters in differentiating low- from high-grade HCC. Moreover, a combination of both techniques further improved the diagnostic performance, suggesting a complementary effect between APT and IVIM imaging. These findings indicate that APT imaging may be a potential noninvasive biomarker for the prediction of histologic grading of HCC and can provide helpful quantitative MR imaging information to assist in HCC diagnosis and clinical treatment strategy. In the future, large-scale investigations are needed to confirm the value of APT imaging in HCC diagnosis and grading.

## Data Availability Statement

The raw data supporting the conclusions of this article will be made available by the authors, without undue reservation.

## Ethics Statement

The studies involving human participants were reviewed and approved by the institutional review board of the First Affiliated Hospital of Xinxiang Medical University. The patients/participants provided their written informed consent to participate in this study. Written informed consent was obtained from the individual(s) for the publication of any potentially identifiable images or data included in this article.

## Author Contributions

BW, FJ, and DH contributed to the conception and design of the study. BW and FJ contributed to paper searching. FJ, XL, and KW contributed to investigation. BW and FJ contributed to data analysis. BW, FJ, and DH contributed to the interpretation of data. BW, FJ, and DH contributed to the drafting of the manuscript, while all authors made critical revision of the manuscript for important intellectual content and gave final approval of the version to be published. BW obtained funding to support this work. BW, FJ, and DH had full access to all the data of the study and take responsibility for the integrity of the data and the accuracy of the data analysis. All authors contributed to the article and approved the submitted version.

## Conflict of Interest

KW was employed by the company GE Healthcare. The remaining authors declare that the research was conducted in the absence of any commercial or financial relationships that could be construed as a potential conflict of interest.
